# A Practical Comparison of Beam Shuttering Technologies for Pulsed Laser Micromachining Applications

**DOI:** 10.3390/ma15030897

**Published:** 2022-01-25

**Authors:** Damon G. K. Aboud, Michael J. Wood, Gianluca Zeppetelli, Nithin Joy, Anne-Marie Kietzig

**Affiliations:** Department of Chemical Engineering, McGill University, Montreal, QC H3A 0C5, Canada; damon.aboud@mcgill.ca (D.G.K.A.); michael.wood3@mail.mcgill.ca (M.J.W.); gianluca.zeppetelli@mail.mcgill.ca (G.Z.); nithin.joy@mail.mcgill.ca (N.J.)

**Keywords:** pulsed laser, laser micromachining, mechanical shutter, solenoid shutter, electro-optic modulator shutter, opening time, lifetime

## Abstract

In this report we investigate the performance of various beam shutter technologies when applied to femtosecond laser micromachining. Three different shutter options are considered: a mechanical blade shutter, a bistable rotary solenoid shutter, and an electro-optic modulator (EOM) shutter. We analyzed the behavior of each shutter type during repeated open/close commands (period of 10 ≤ *T* ≤ 200 ms) using both high-speed videography and practical micromachining experiments. To quantify the performance at varying cycle periods, we introduce a new variable called the compliance that characterizes the average state of the shutter with respect to its intended position. We found that the solenoid shutter responds poorly to sequential commands. The mechanical shutter provides reliable performance for cycled commands as short as *T* = 40 ms, but begins to lag significantly behind the control signal for *T* ≤ 20 ms. The EOM shutter provides the most precise and reliable performance, with an opening time of only 0.6 ms and a high compliance with the signal commands, even when cycled very quickly (*T* = 10 ms). Overall, this study acts as an extensive practical guide for other laser users when considering different shutter options for their laser system and desired application.

## 1. Introduction

The beam shutter is a crucial component of a laser micromachining system. However, it is often rarely or only roughly mentioned in research studies. The ability to accurately control when a surface is exposed to the laser beam allows for the fabrication of intricate microstructures and geometrically well-defined surface textures. Having control over the exact number of pulses irradiating the surface ensures uniformity of the surface textures or structures being ablated.

Over the past twenty years, researchers have used several different shuttering technologies such as electromechanical shutters, electromagnetic shutters, and electro-optical shutters. For example, electromechanical shutters have been applied in various applications, such as the development of superamphiphobic patterns on PTFE [1], the fabrication of conical spike arrays on silicon [2], and a fundamental study of the threshold fluences and incubation coefficients of different metals [3]. Electromagnetic shutters have been applied in the biomimetic surface texturing of metals [4] and the formation of periodic surface structures on dielectric materials [5]. An electro-optical shutter was employed to study the ablation threshold and damage morphology of common metals [6]. Further, characterization studies have also been conducted on electro-optical shutters such as optical Kerr gates [7]. Some examples of recent advances in shutter technology include the reduction in vibrations of electromechanical shutters [8,9], integration of photodiodes into mechanical shutters [10], and the fabrication and characterization of an optical shutter based on a piezoelectric cantilever [11].

**Figure 1:**
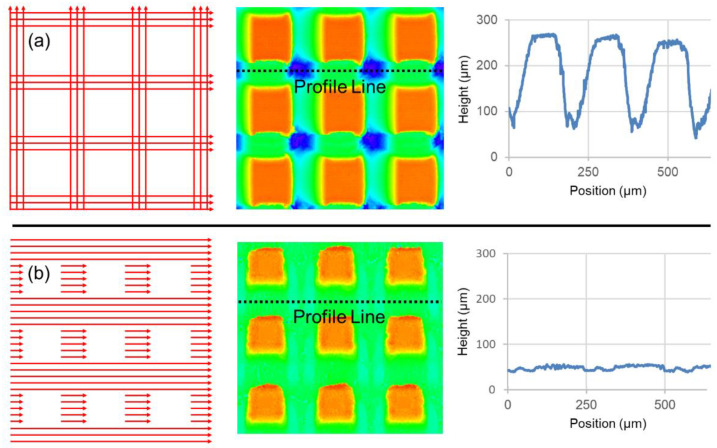
Comparison of square pillar microstructures fabricated using a simple grid pattern without a shutter (**a**) and the same structures fabricated using a sophisticated shutter system, allowing for greater control of the accumulated ablation depth (**b**).

Without an effective shuttering system, laser texturing applications are severely limited. For example, as shown in Figure 1a, it is possible to micromachine an array of square pillars without a shutter, whereas long, overlapping lines can be intersected in the x- and y-directions, producing a grid. However, as demonstrated by the topographical heat map and corresponding cross-sectional profile line, this method of generating surface textures causes some portions of the surface to receive twice the amount of laser pulses. The unavoidable side effect of this approach is the creation of some areas which are deeper than others along with differing local surface chemistries. This problem of overlapping fluence profiles with uncontrolled depth has been witnessed in numerous applications, e.g., wettability studies on ablated gridlike structures [12,13] and dynamic studies concerning the influence of micromachining on droplet mobility and splashing [14,15].

In contrast, Figure 1b shows an example of the same intended, inscribed microstructure now realized using a beam shuttering device to allow for dead zones in the raster scan while only machining in the x-direction. This added component makes it possible to micromachine structures with a far greater level of control—in this case, the fabrication of square pillars arrayed within flat valleys of even depth.

In this report, we investigate the performance of three different shuttering options: a mechanical blade shutter, a bistable rotary solenoid shutter, and an electro-optic modulator (EOM). The mechanical and solenoid shutters function by blocking the light using a physical blade which is moved mechanically into or out of the beam path. In contrast, EOM shutters function using electro-optical devices such as Pockels cells, which shutter the laser beam electromagnetically. Having a deeper understanding of what shutter systems are available for various applications can assist the user in making informed decisions on what technology to use. Hence, the goal of this report is to provide a practical comparison for laser system users of three different beam shutter technologies.

## 2. Materials and Methods

### 2.1. Micromachining Setup

The micromachining setup used in this work consists of a Libra Ti:Sapphire laser system (Coherent, Inc., Santa Clara, CA, USA) with a central wavelength of 800 nm, pulse duration <100 fs, and a 1 kHz repetition rate. For this work, the pulse energy was set at 100 µJ and a spot size of 8 µm was used, corresponding to a pulse peak fluence of 398 J/cm^2^. This irradiation source is complemented with a sample positioning system consisting of XY linear translation stages which are actuated by an XPS universal high-performance motion/driver controller (Newport Corp. Irvine, CA, USA). This same motion/driver controller outputs a transistor–transistor logic (TTL) signal to signify the open/close position of a beam-blocking shutter in conjunction with the stage movements. All axis movements, including stages and beam shuttering, are user-defined by position–velocity–time trajectory tables, where each axis adheres to a list of positions specified in said table at defined times with a defined running velocity for each position point.

### 2.2. Shuttering Methods

#### 2.2.1. Mechanical Shutter

**Figure 2:**
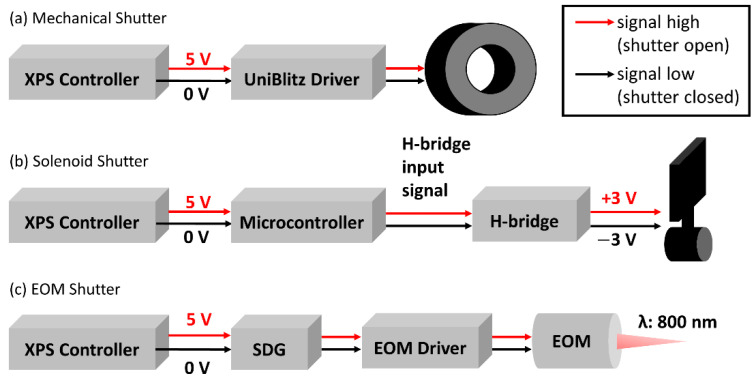
Box chart visualizing the signal pathway for each shutter. (**a**) Mechanical shutter. (**b**) Solenoid Shutter. (**c**) EOM shutter.

We included in our tests a widely used, commercially available shutter—the unistable Uniblitz VS25 optical shutter (Vincent Associates, Inc., Rochester, NY, USA). This unit has an aperture of 25 mm and two shutter blades which have a specified opening time of 6 ms. This shutter interfaces with the *XPS* through a VCM-D1 shutter driver (Vincent Associates). Figure 2a presents a box chart visualization of the signal pathway for this shutter.

#### 2.2.2. Solenoid Shutter

We next included a simple solenoid shutter in our tests—the bistable *BOS7/10* rotary optical shutter (Takano Co., Ltd., Chiyoda City, Tokyo, Japan). This unit has a 12.3 mm × 10.3 mm shutter blade which rotates over an operating angle of 50° and has a quoted response time of <17 ms. A permanent magnet ensures that the shutter remains stationary at either extreme of this rotating path when the solenoid is de-energized. The shutter is actuated to rotate to either its open or closed position by the application of positive or negative 3 V. As shown in Figure 2b, we programmed an Arduino microcontroller to read the TTL signal from the *XPS* and activate a H-bridge to handle this voltage switching (See Supporting Note S1 for further details).

#### 2.2.3. Electro-Optic Modulator Shutter

Finally, we included an electro-optic beam shuttering method in our tests. Ultrafast laser systems achieve useful power through Chirped Pulse Amplification, whereas a stretched seed beam is passed multiple times through a solid-state laser gain medium to gain the power of a pump beam. In our case, a *QX-1020* KD*P electro-optic shutter (Gooch & Housego, PLC, Ilminster, UK) with a rise-fall time of 800 ps controls entry of the seed beam to the regenerative amplifier cavity containing the gain medium. This electro-optic shutter is actuated by an SDG Elite synchronization and delay generator (Coherent, Inc., Santa Clara, CA, USA) which can in turn be gated using an external TTL signal. As shown in Figure 2c, the TTL signal from the XPS is used to shutter the laser seed beam, resulting in controlled laser beam output from the Libra (see Supporting Note S2 for further details).

#### 2.2.4. Measurement of Shutter Action

**Figure 3:**
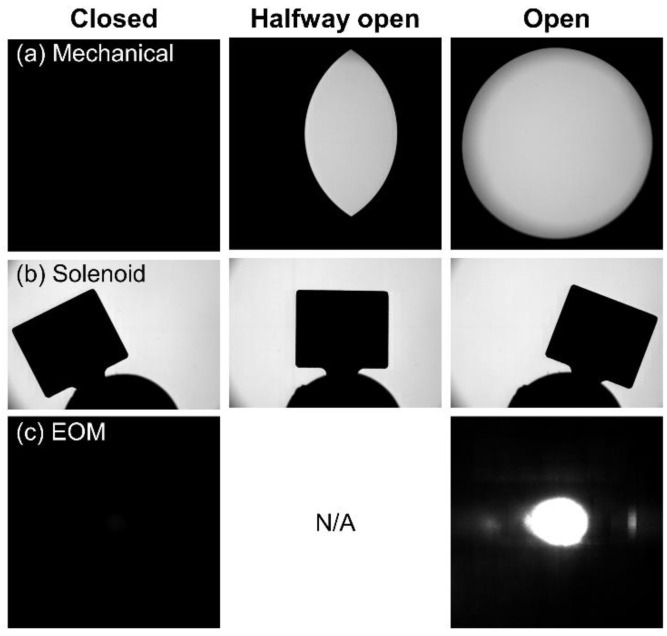
Frames of high-speed video footage used to measure the position of each shutter over time. (**a**) Mechanical shutter. (**b**) Solenoid Shutter. (**c**) EOM shutter.

To accurately measure the position of each shutter tested in this report, we used a *FastCam SA5* high-speed camera (Photron USA, Inc., San Diego, CA, USA) equipped with a *Zoom 7000* 18–108 mm macro zoom lens (Navitar, Inc., Ottawa, ON, Canada) to observe the shuttering action in real time at 2000 to 5000 frames per second. Figure 3 displays representative snapshots of each shutter in the closed, halfway open, and fully open states. Note that for the EOM shutter, there is no observable physical component. Therefore, we recorded the intensity of light on a surface at which the laser beam was aimed. Since the pulse duration of the laser is on the order of femtoseconds, the pulses occur on a timescale approximately 10^12^ times faster than the camera frame rate and hence no intermediate intensities are recordable. As a result, our experimental setup does not allow for the recording of a frame in which this shutter is halfway open. Supporting Videos S1–S3 show the original videos from which the snapshots in Figure 3 were obtained.

### 2.3. Substrate Material

All micromachining experiments were performed on a single, polished coupon of P20 tooling steel purchased from McMaster Carr (Elmhurst, IL, USA). This material has a chemical composition of: Fe (94.82%), C (0.38%), Si (0.30%), Mn (1.37%), S (0.002%), Cr (1.95%), Ni (1.00%), Mo (0.18%).

## 3. Results

**Figure 4:**
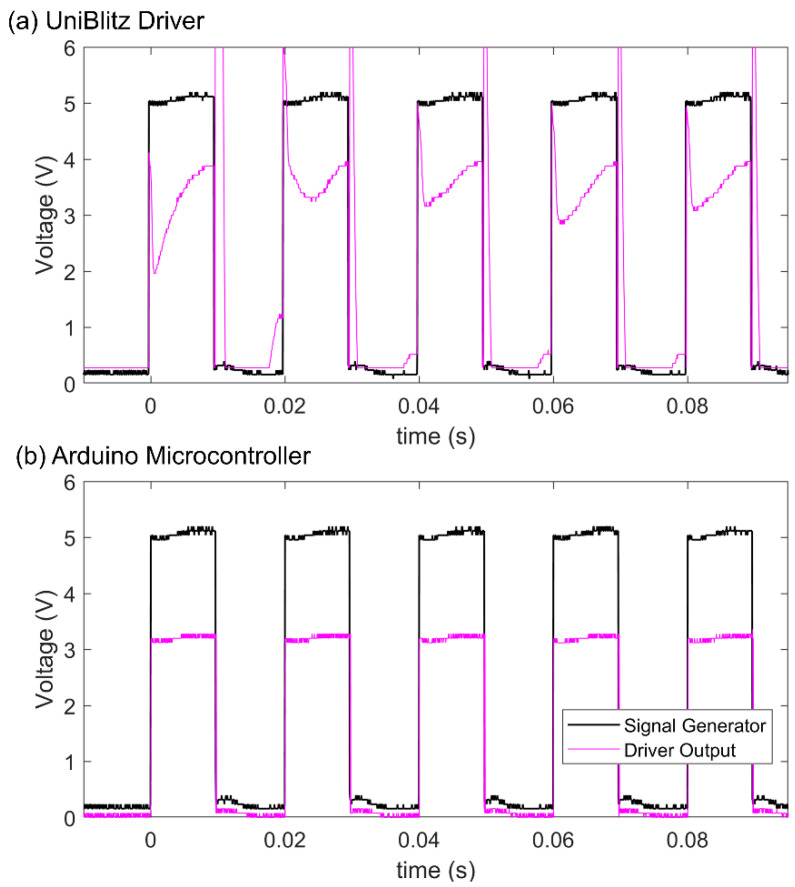
Simultaneous oscilloscope readings of the XPS controller signal and the: (**a**) Uniblitz driver output signal to the mechanical shutter, and (**b**) Arduino microcontroller output signal to the EOM and solenoid shutters.

We began our work with tests of possible delays introduced by the electronic control equipment in the signal pathway. That is, we took simultaneous oscilloscope readings of the *XPS* controller TTL signal and the signal sent forth by the UniBlitz driver or Arduino microcontroller, with a temporal resolution of 0.04 ms. As shown in Figure 4, neither the commercial mechanical shutter driver nor the microcontroller we programmed ourselves to activate the H-bridge introduce any measurable delay in the electrical signaling of their respective shutters. There is no offset in the square high/low TTL signal and the respective square high/low shutter driver signals. These findings tell us that any deviation from ideal shutter behavior will be the result of mechanical—not electrical—limitations of the systems.

**Figure 5:**
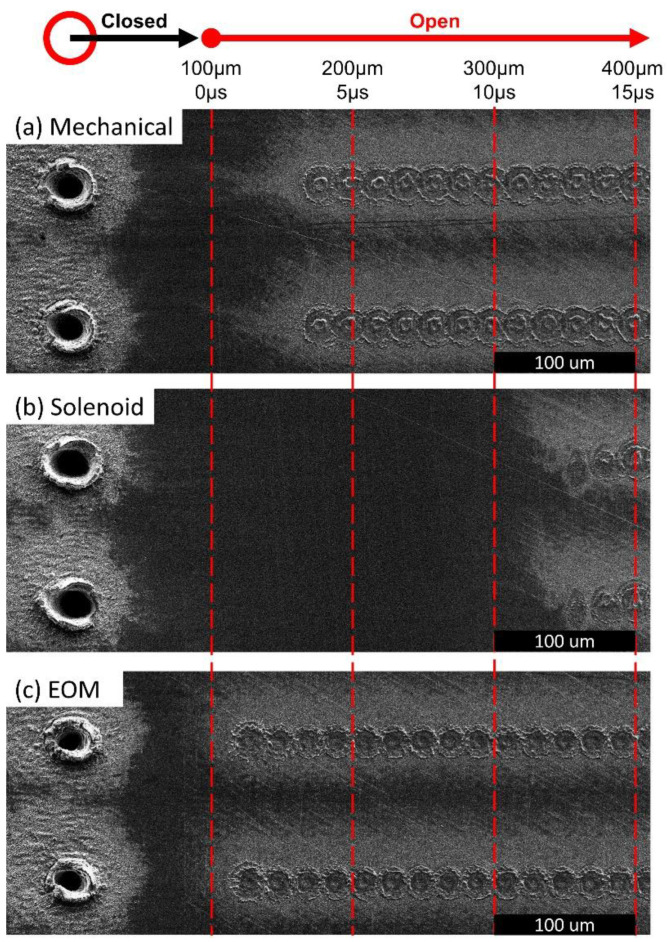
SEM micrographs demonstrating how the opening time of each shutter was measured. The opening time can be discerned based on the distance of the first ablating pulse from the deep hole on the left. (**a**) Mechanical shutter. (**b**) Solenoid Shutter. (**c**) EOM shutter.

The practical opening time of each different shutter type was next tested using a laser micromachining experiment. As shown by the illustration at the top of Figure 5, a hole was first drilled on the left side of the sample. Next, the shutter was closed, and the beam position began shifting with a translational velocity of 20 mm/s towards the right. Finally, after 5 ms (at a distance of exactly 100 µm), the shutter was signaled to open while the beam continued to shift across the surface. This test was repeated eight times for each shutter. Based on this simple experiment, the practical opening time can be elucidated in Figure 5 from the distance between the large hole on the left and the first discernible ablated spot to the right. For example, in the case of the mechanical shutter we observe an average distance of 170.5 ± 4.4 µm, corresponding to a practical opening time of 3.03 ± 0.22 ms. For the solenoid shutter, we find that *t_open_* = 12.20 ± 0.29 ms, and for the EOM shutter we find that *t_open_* = 0.62 ± 0.22 ms.

**Figure 6:**
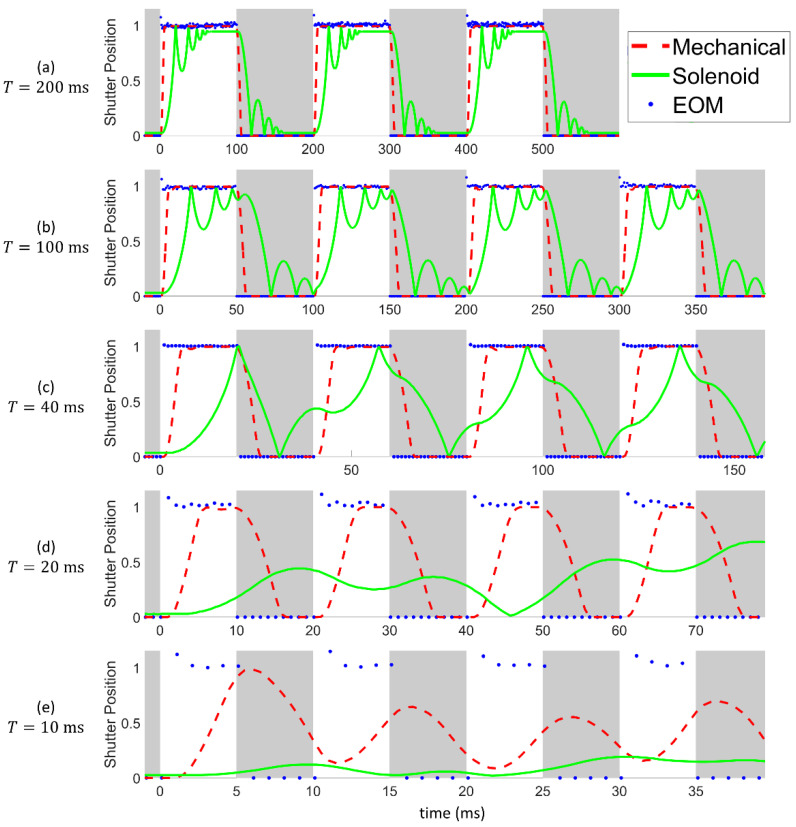
Measured position of each shutter over time for different cycling periods. (**a**) *T* = 200 ms. (**b**) *T* = 100 ms. (**c**) *T* = 40 ms. (**d**) *T* = 20 ms. (**e**) *T* = 10 ms.

Next, we consider the performance of each shutter while responding to a long sequence of open/close commands. Figure 6a graphs the measured position of each shutter for a period of *T* = 200 ms (i.e., 100 ms open, 100 ms closed, repeating). In this figure, the grey shaded blocks represent times where the shutter is commanded to be closed, and the white shaded blocks represent open segments. Meanwhile, the actual shutter position is represented by a number ranging from zero (completely closed) to one (completely open). It is clear that both the mechanical and the EOM shutter respond nearly perfectly to the signal commands at this frequency, while the solenoid shutter has a noticeable time lag. In addition, the position of the solenoid shutter oscillates several times while switching to a different state. This occurs due to the physical momentum of the solenoid shutter’s blade, which bounces back slightly towards the center position after reaching the intended open/closed position. Hence, it takes approximately 50 ms for this shutter type to come to rest in the intended position after it first arrives there. This problem can be easily visualized in Supporting Video S2, which shows a high-speed video of the solenoid shutter in action.

Figure 6c graphs the measured positions of the shutters for *T* = 40 ms. Here, we see that the opening time of the mechanical shutter is clearly visible, since the dashed red line representing the mechanical shutter appears to rise and fall much later than the change in the command signal. However, at this cycling period, the solenoid shutter is no longer able to keep up with the speed of the commands. As explained above, the solenoid shutter requires approximately 50 ms to come to rest once it reaches the intended position, during which time it continues to bounce at the end of its movement path. Hence, when a new command signal is received before this oscillation period is complete, the shutter blade begins to act quite uncontrollably, bouncing back and forth rapidly between the extremes of its movement path. This oscillating behavior of the solenoid shutter’s blade illuminates another important consideration when selecting a shutter for laser processes: for shutters that rely on the movement of physical components, the opening time of the shutter is not sufficient to characterize its performance. For example, based on the solenoid shutter’s measured opening time of *t_open_* = 12.20 ± 0.29 ms, some users might assume that this shutter would perform adequately at a cycling period of *T* = 40 ms. However, Figure 6c clearly shows that at this cycling period, the momentum of the shutter blade causes serious performance problems. Therefore, when considering different shutter options for laser micromachining applications, the influence of the momentum of the shutter’s blades should be accounted for. In this specific case, the slow response of the solenoid shutter can be attributed to its operating voltage. The BOS7/10 solenoid used in this study can operate at a voltage ranging from 3–12 V. At higher voltages, the shutter actuates more quickly, but the duty cycle of the device is decreased. Hence, if the solenoid is used at a 100% duty cycle with a higher operating voltage it will overheat, causing the internal resin bobbin to melt, which can lead to a short circuit in the coil. Therefore, for this type of shutter, there is an inherent tradeoff between the performance of the device and its lifetime. We operated the solenoid shutter at a voltage of 3 V, at which the response time is relatively slow, but the shutter can be operated at any duty cycle.

Figure 6d,e presents our observations for *T* = 20 and 10 ms. At these very fast cycling times, the solenoid shutter no longer functions at all, reaching shutter positions no greater than 0.22 in the graph. At such low shutter positions, the edge of the shutter’s blade does not move far enough for even the periphery of the laser beam to pass. From a practical perspective, the solenoid shutter remains closed throughout the entire experiment. The mechanical shutter still performs relatively well at *T* = 20 ms, faithfully following the command signal with only a short lag time of 3 ms. However, at *P* = 10 ms (Figure 6e), this shutter finally fails. This failure was expected at this cycling period since the manufacturer states that the shutter has an opening time of 3–6 ms, and hence a cycling time of 6–12 ms, which matches the duration of the signal tested. In contrast, the EOM shutter continues to perform nearly perfectly throughout every cycling period tested. Even at a cycling period of only *T* = 10 ms, the EOM shutter is almost always in the correct open/closed position. Only a few datapoints represent positional errors wherein the shutter is open during a close command or closed during an open command.

**Figure 7:**
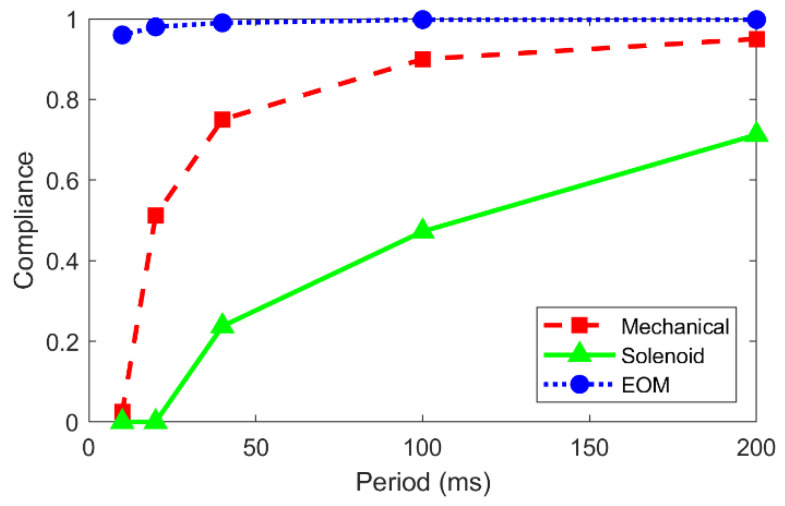
Compliance versus cycling period for each shutter.

To quantify the performance of the different shutters at various cycling periods, we introduce a new parameter that we call the compliance, C, which is calculated based on the shutter’s position during the *open* signals sent by the controller. So if the total duration of *open* signals sent by the controller is given by *t_open,signal_*, and the measured time that the shutter is actually open (during the *open* signal) is *t_open,meas_*, then *C* = *t_open,meas_*/*t_open,signal_*. Using this definition, the compliance is graphed versus the cycling period in Figure 7. Clearly, each shutter performs best at longer cycling times. For example, at *T* = 200 ms, the values are *C_EOM_* = 1, *C_mech_* = 0.95, and *C_sol_* = 0.71, whereas for *T* = 10 ms, the values are as low as *C_EOM_* = 0.96, *C_mech_* = 0.02, and *C_sol_* = 0. This graph also serves as a clear visualization that in general, the EOM shutter is the highest performing option, and the solenoid shutter has the worst performance.

**Figure 8:**
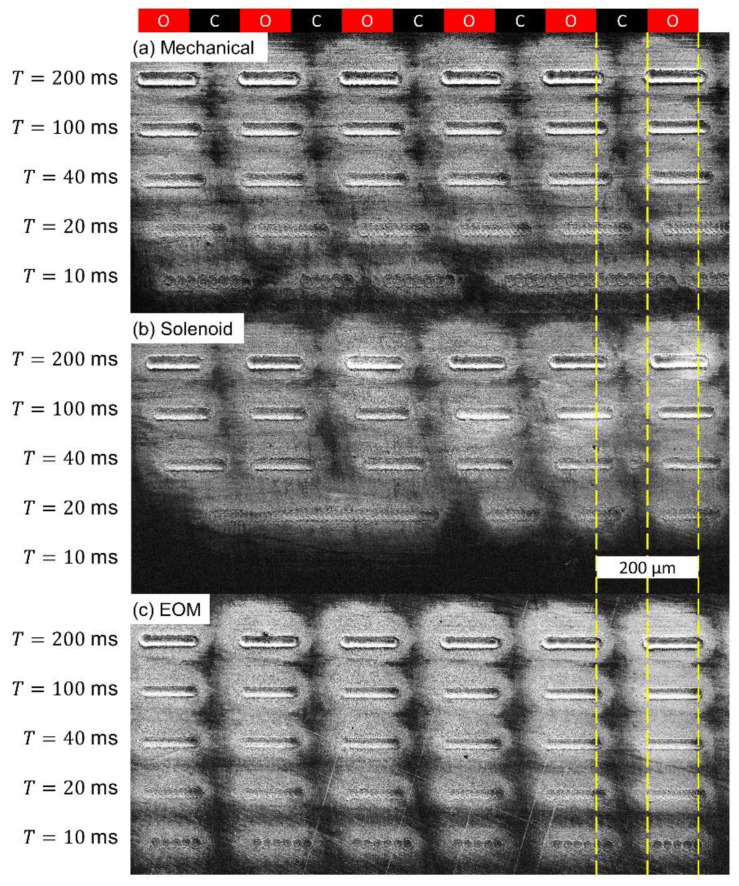
SEM micrographs demonstrating the performance of different shutters when subjected to a cycling open/closed signal with varying period. Ideally, each ablated line should be 100 µm long, and remain aligned with those above it. (**a**) Mechanical shutter. (**b**) Solenoid Shutter. (**c**) EOM shutter.

Figure 8 provides a physical demonstration of the conclusions drawn from Figure 6 and Figure 7. For each shutter, a series of horizontal dashes are micromachined at various cycling times. The translation speed used for each line is adjusted such that the length of each dash, and the spaces between them, are intended to be 100 µm (as shown by the yellow lines). Accordingly, dashes at shorter cycling periods are machined at a faster translation speed. The red and black boxes at the top of the figure indicate the horizontal sections in which the shutters should be open (O) and closed (C). These images confirm the trends observed in Figure 7. That is, for long cycling times all three shutter options successfully execute the signal instructions. Additionally, as the cycling period is reduced, the dashes begin to lag behind the signal instructions. The mechanical shutter exhibits noticeable lag times at *T* = 20 ms and fails completely at *T* = 10 ms. The solenoid shutter exhibits noticeable lag times throughout the entirety of the experiment and fails completely at *T* = 20 ms. The EOM shutter performs extremely well at each cycling period tested, only showing noticeable lag behind the command signal when *T* = 10 ms.

**Figure 9:**
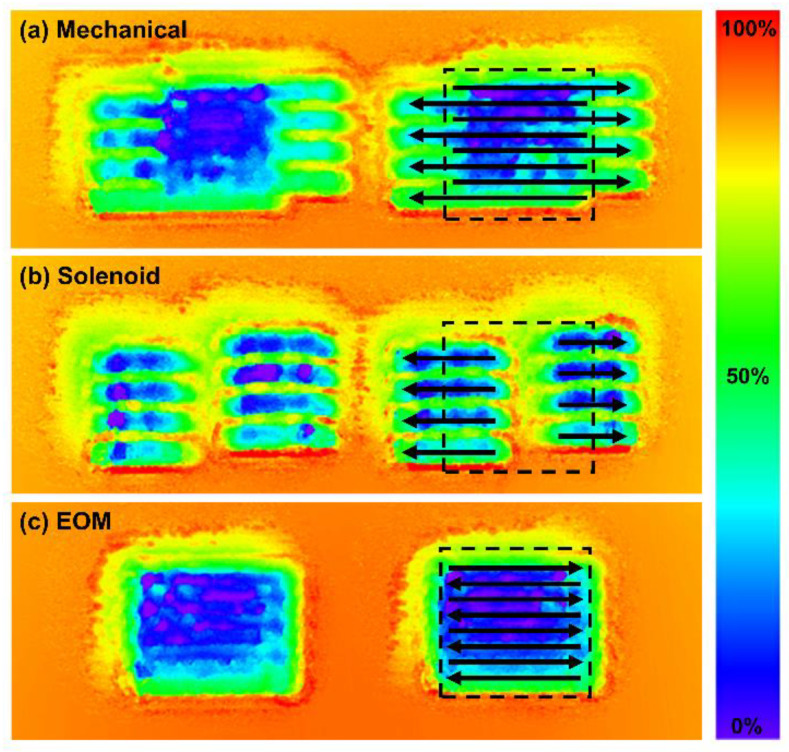
Heatmaps visualizing the topography of ablated microstructures on steel. As shown in the bar on the right side of the image, red colors correspond to high sections of material, and blue sections correspond to low sections. (**a**) Mechanical blade shutter. (**b**) Bistable rotary solenoid shutter. (**c**) EOM shutter.

As a final test of each shutter technology considered in this report, we attempted to micromachine an array of 100 × 100 µm^2^ square holes, spaced 100 µm apart. The beam was raster scanned across the surface with a translation speed of 5 mm/s, corresponding to an open/close time of 20 ms, and hence a cycling period of 40 ms when considering a wide array of inscribed features. Figure 9 displays topographical heat map images of the results, so that the depth of the features can be visualized. Figure 9a shows the features inscribed by the mechanical shutter, which are clearly affected by the lag time of this device. On the right feature, a dashed square box illustrates the intended shape of the square, and the arrows indicate the direction of the raster scanning beam. It is clear that the lag time of the shutter causes each pass of the beam to be displaced slightly from the previous, resulting in misaligned edges of the microstructure. This effect is even more clearly demonstrated by the solenoid shutter (Figure 9b), where subsequent passes of the beam do not overlap at all. The magnitude of this problem can actually be predicted by the solenoid shutter’s measured opening time of 12.20 ms. Considering the translation velocity of 5 mm/s, the opening time of the shutter leads to a displacement of 61 µm in each direction, such that subsequent lines, when the beam is raster scanned left and right, become misaligned by a total of 122 µm—greater than the width of the intended microstructures. Therefore, it is not possible for this shutter to inscribe overlapping lines to develop features smaller than 122 µm.

Figure 9c represents the EOM shutter, which has a clear square shape resulting from the low lag times and high compliance of this shutter technology. In this case, misalignment of consecutive raster scan passes is only noticeable from the wavy edges on the left and right sides of the square holes.

The misalignment issue demonstrated in Figure 9 stemming from the back-and-forth relative motion of the beam can indeed be avoided if a raster scanning trajectory is not used. Rather, the laser trajectory can be designed to exclusively open the shutter during rightwards passes. In this scenario, the delay caused by the shutter leads to a relative horizontal displacement of the beam (and hence micromachined features) in a single direction. However, this possibly increases the processing time required to ablate the surface, since half of the beam’s trajectory is not being utilized for machining—a consideration of importance especially for slow translation systems. Therefore, perfect alignment of the beam during a conventional back-and-forth raster scan is desirable whenever possible.

All of the figures above prove that the EOM shutter considered in this report demonstrates the highest performance in every metric tested: the opening time, consistency during cycling, overall compliance, and practical laser machining of microstructures. These comparisons are summarized in Table 1. For the sake of comparison, Table 1 also provides technical details about several other shutter options not tested in this report. The information in Table 1 was gathered both from publicly available datasheets on the manufacturers’ websites, and from personal communications with the manufacturers. Supporting Note S3 provides screenshots and website links to the datasheets. Comparing the data in Table 1, it is noticeable that the solenoid shutters are the most cost-effective, but they have very long opening times. Hence, solenoid shutters are appropriate only for applications where a short opening time is irrelevant. Unfortunately, since EOMs are not generally marketed as shutters, we were unable to obtain the same comparative information (such as lifetime and cost) for that shutter type. However, in Supporting Note S2 we have listed the names and specifications of some Pockels cells (and drivers) available on the market today, which could be used as shutters.

Considering the application of our findings to the laser micromachining field today, it should be noted that in general, most industrial-grade, high-frequency, ultrashort pulsed laser systems already utilize an EOM for beam shuttering [6,7]. This is necessary to accommodate the high scanning speeds and high repetition rates required to machine products in a time-efficient manner. However, in more fundamental academic research, many facilities rely on low-frequency pulsed lasers which are often shuttered using conventional electromechanical devices. Yet, in many cases, the purchase of an electromechanical shutter is unnecessary, since an EOM is already integrated into the laser’s regenerative amplifier cavity but is simply not being used to its full potential as a shutter for micromachining applications [12]. Therefore, integrating EOM shuttering into many laser micromachining setups would have no associated cost.

## 4. Conclusions

In this report, we compared the performance characteristics of three different shutter options: a mechanical blade shutter, a bistable rotary solenoid shutter, and an electro-optic modulator.

When each shutter was tested with a cycled on/off signal at various frequencies, we observed that the momentum of the shutter’s physical components can influence the shutter’s response to subsequent signal pulses, leading to unreliable actuation and reduced compliance. Therefore, the opening time of a shutter is doubly important when responding to a long series of on/off commands, in which a slow response to one command leaves the shutter’s physical barrier still in motion when the next command is received. This effect was most prominent for the solenoid shutter, which was observed oscillating its position at the end of its movement path.

Comparing the prices and performance of each shutter, the solenoid shutter is the most affordable option, but has very poor performance in terms of the opening time and overall compliance. Therefore, solenoid shutters should only be considered in applications with cycling times greater than 100 ms and where a slow opening time is not detrimental.

In contrast, the EOM demonstrated the best performance in every category tested: the opening time, consistency during cycling, overall compliance, and practical laser machining of microstructures. Therefore, for laser systems which rely on chirped pulse amplification within a regenerative amplifier cavity, the EOM should be applied as the beam shuttering method, since the optical components are already present. Furthermore, for other types of laser systems that are not based on a regenerative amplifier cavity, an EOM could still be considered as an effective, albeit pricier, shuttering system by installing it outside of the laser system itself.

## Figures and Tables

**Table 1:** Comparison of cost and performance characteristics of shutters. In the left column, the shutter models in bold font are the ones that were empirically tested in this report. Values marked with an asterisk (*) were measured experimentally. All other values are obtained from the manufacturers.

Shutter Name	Type	Opening Time [ms]	Lifetime [Cycles]	Compliance (T = 40 ms)	Compliance (T = 200 ms)	Cost [USD]
**Vincent Associates—CS45**	Electromechanical	3.03 ± 0.22 *	1.0 × 10^6^	0.75 *	0.95 *	705–1570
ThorLabs—SHB025	Electromechanical	3	1.5 × 10^7^			962
NM Laser Products—LST200SLP	Electromechanical	1	>10^9^			495
**Takano—BOS 7/10**	Solenoid	12.20 ± 0.29 *	5 × 10^6^	0.24 *	0.71 *	145
Brandstrom Instruments (Custom)	Solenoid	25–35	2.5 × 10^6^			250–350
Ellis/Kuhnke Controls—CDR030	Solenoid	30	2 × 10^7^			370
**Gooch & Housego Model QX Pockels Cell**	EOM	0.62 ± 0.22 *	Not Available	0.99 *	1.00 *	Not Available

## Data Availability

The data presented in this study are available on request from the corresponding author.

## Supplementary Materials

The following are available online at https://www.mdpi.com/article/10.3390/ma15030897/s1, Note S1: Solenoid Shutter, Note S2: Signal Delay Generator, Table S1: List of some Pockels cells available on the market, Table S2: List of some Pockels cell drivers available in the market, Note S3: Screenshots and Links for Information on Shutter Specifications and Cost, Video S1: high-speed video of mechanical shutter responding to open/close commands with a cycling period of 100 ms, Video S2: high-speed video of solenoid shutter responding to open/close commands with a cycling period of 100 ms, Video S3: high-speed video of EOM shutter responding to open/close commands with a cycling period of 100 ms.
