# Supplementary material for: A Practical Comparison of Beam Shuttering Technologies for Pulsed Laser Micromachining Applications
Source: Materials (Basel). 2022 Jan 25;15(3):897. doi: 10.3390/ma15030897 (PMC8839459; doi:10.3390/ma15030897)
Supplement: Supplementary file 1 [file materials-15-00897-s001.zip › Supplementary Materials.pdf]

Supplementary Materials

Comparison of beam shuttering techniques for pulsed laser micromachining applications

Damon G. K. Aboud, Michael J. Wood, Gianluca Zeppetelli, Nithin Joy and Anne-Marie Kietzig *

Department of Chemical Engineering, McGill University, Montreal, QC H3A 0C5, Canada; damon.aboud@mcgill.ca (D.G.K.A.); michael.wood3@mail.mcgill.ca (M.J.W.); gianluca.zeppetelli@mail.mcgill.ca (G.Z.); nithin.joy@mail.mcgill.ca (N.J.)

* Correspondence: anne.kietzig@mcgill.ca

Supporting Note S1. Solenoid Shutter

Wiring Diagram

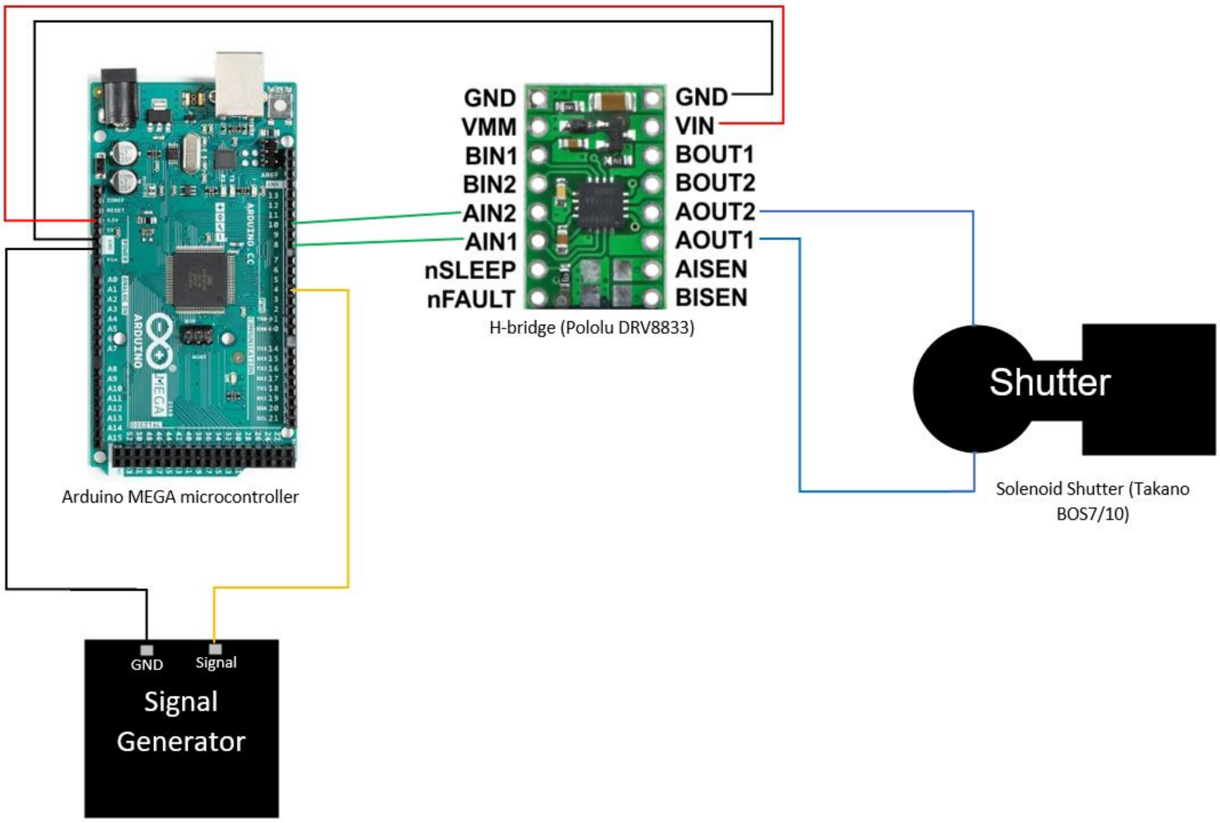

H-bridge details

An H-bridge is an electronic circuit with both inputs and outputs that allows the polarity of the voltage applied on a load to be switched (in this case the load is the solenoid shutter). The H-bridge switches polarity based on the inputs given to it. The *Arduino* microcontroller and the H-bridge can be configured in such a way that when the microcontroller reads a high signal, the shutter will rotate in one direction. Similarly, when the microcontroller reads a low signal, the shutter will rotate in the opposite direction.

Arduino Code

```
// Pin Assignments
int XPS_Signal_Pin = 40;
int Solenoid_A1_Pin = 7;
int Solenoid_A2_Pin = 9;

// General Variables
int Last_State = 0;
int Current_State = 0;


void setup() {
  pinMode(XPS_Signal_Pin, INPUT);
  pinMode(Solenoid_A1_Pin, OUTPUT);
  pinMode(Solenoid_A2_Pin, OUTPUT);
  Last_State = digitalRead(XPS_Signal_Pin);
}

void loop() {
  Current_State = digitalRead(XPS_Signal_Pin);
  if(Current_State != Last_State) {
    State_Change();
  }
}
```

```
void State_Change(){
  digitalWrite(Solenoid_A1_Pin, Current_State);
  digitalWrite(Solenoid_A2_Pin, Last_State);
  Last_State = Current_State;
}
```

Supporting Note S2. Signal Delay Generator

An electro-optic modulator (EOM) is essential for a femtosecond laser system that has a regenerative amplifier. The regenerative amplifier is a device used to amplify ultrashort pulses – those on the order of picoseconds or femtoseconds. EOMs can be controlled externally and are used to pick up pulses at desired intervals. In the present work, the electro-optic shutter is controlled by a synchronization and delay generator (SDG) which sends out commands to the electro-optic modulator placed inside the regenerative amplifier cavity of the femtosecond laser system. Our electro-optic modulator is a KD*P Pockels cell whose operating range is 300-1100 nm with a rise-fall time of 800 ps.

Table S1. List of some Pockels cells available in the market.

Manufacturer	Series/Model	Wavelength (nm)	Crystal Type	Quarter-wave Voltage (kV)
Gooch & Housego	QX	300 - 1100	KD*P	Varies
Eskma Optics	D-mini/8-800	800	KD*P	<2.7
Inrad Optics	PKC	250 - 1320	KD*P	3.3

Table S2. List of some Pockels cell drivers available in the market.

Manufacturer	Series/Model	Output Voltage (kV)	Rise/Fall time (ns)	Repetition Rate (kHz)
Gooch & Housego	Q-Drive	1.5 – 4.5	4 - 7	0.0 – 4.5
Eskma Optics	DP-SP	Up to 3.6	< 6 - 7	Up to 600
Inrad Optics	-	2.4 to 6.0	≤ 20	Up to 50

Supporting Note S3. Screenshots and Links for Information on Shutter Specifications and Cost

Vincent Associates – CS45 Cost

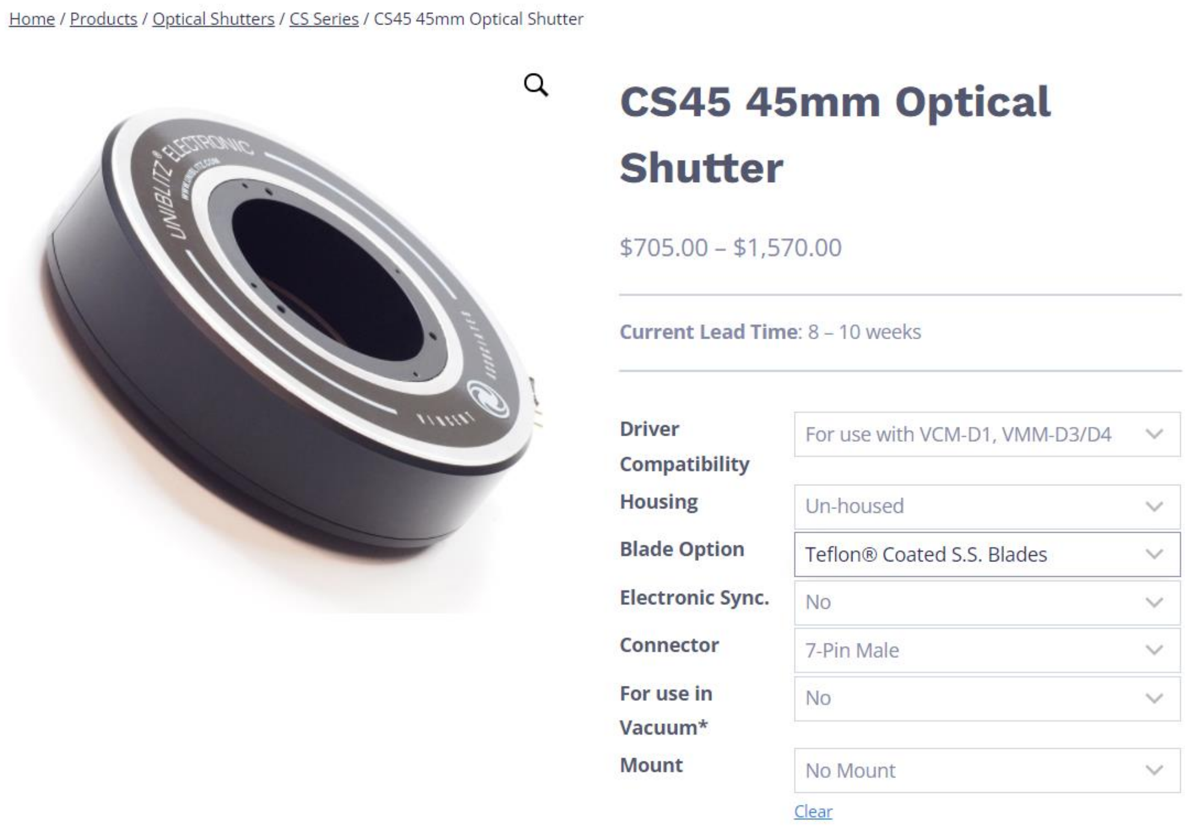

ThorLabs – SHB025 Specifications and Cost

Item #		SHB025(T)		SHB05(T)		SHB1(T)	
**Operating Frequencies**							
Operating Frequency Range		0 - 50 Hz		0 - 25 Hz		0 - 15 Hz	
Max Operating Frequency, Protected Mode[b]	1 Minute	16 - 50 Hz[d]		16 - 25 Hz[d]		-	
	10 Minutes	-		-		14 - 15 Hz	
	30 Minutes	-		-		11 - 13 Hz	
	Continuous[c]	15 Hz		15 Hz		10 Hz	
Max Operating Frequency, Unprotected Mode[b]	Continuous[c]	50 Hz[d]		25 Hz[d]		15 Hz	
**Key Timings**		Typical	Max	Typical	Max	Typical	Max
Minimum Exposure Pulse[e]		10 ms	12 ms	29 ms	31 ms	28 ms	30 ms
Rise (Open) Time		3 ms	4 ms	8 ms	10 ms	10 ms	12 ms
Fall (Close) Time		3 ms	4 ms	9 ms	12 ms	9 ms	11 ms
Exposure Accuracy[f,g]		2 ± 0.5 ms		2 ± 0.5 ms		6 ± 0.5 ms	
Jitter (Rising or Falling Edge)	≤10 Hz	1.8 ms	4 ms	1.8 ms	4 ms	1.7 ms	4 ms
	10 to 11 Hz					-	-
	11 to 15 Hz					4 ms	8 ms
	15 to 16 Hz	-	-	-	-	N/A	N/A
	16 to 25 Hz	3.5 ms	8 ms	3.5 ms	8 ms	N/A	N/A
	25 to 50 Hz			N/A	N/A	N/A	N/A
Exposure Repeatability Jitter[g,h]		<4 ms					
**General Specs**							
Typical Lifetime		15 Million Cycles					
Operating Temperature		15 to 40 °C					

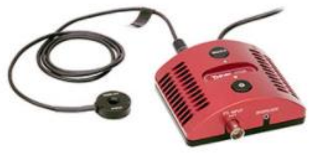

Zoom

Complete Product Details

Part Number: SHB025 -Ask a technical question

Package Weight: 2.65 lbs / Each

Available: 7-10 Days

RoHS: RoHS✓

Price: $961.66

Add To Cart: Qty : 1

Add To Cart

Release Date: Sep 15, 2016

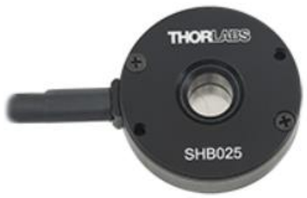

Link to Manual:

https://www.thorlabs.com/drawings/2012f477ef7e3f76-B5C48A4E-E781-15B8-8C3A92B2BAF04915/SHB025-Manual.pdf

NM Laser Products – LST200SLP Specifications and Cost

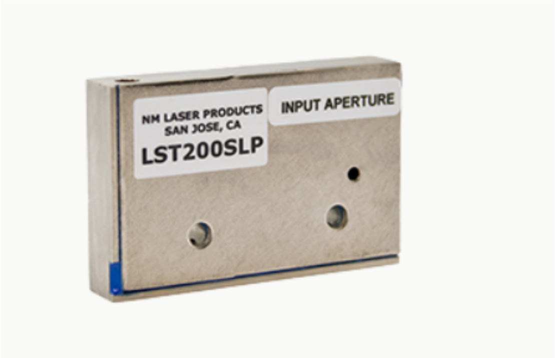

High-Speed Laser Shutter / Optical Shutter – LST200SLP

Features

Optical Power Handling: 2 W

Aperture Size: 2.3 & 3 mm Option

Switching Speed: 1 ms

Lifetime: Billions of Cycles

Price: $495

SUBMIT RFQ

Link to Specifications:

https://www.nmlaser.com/products/lst200slp/

Takano – BOS7/10 Specifications

◆Main Specifications

Heat-Resistant Class	Class E (120℃)
Rated Voltage	3 (V DC)
DC Resistance	9.5 (Ω)
Coil Saturation Temperature Rise $\Delta\theta_s$ (at 20℃)	$\Delta\theta_s \fallingdotseq 59 \times W$ (℃)<br>$K \fallingdotseq 59$ (℃/watt)
Temperature Rise Time Constant τ	1 minute
Mass	4 (g)
Shutter Dimensions	12.3 × 10.3 (mm)
Operating Angle	50 (°)
Life Cycle/Durability	5,000,000 (cycles)
Response Speed – with Applied Voltage 3V DC	18 (ms) or less
Response Speed – with Applied Voltage 5V DC	13 (ms) or less
Response Speed – with Applied Voltage 12V DC	8 (ms) or less

Link to Datasheet:

https://www.takano-sanki21.com/file/sanki/en/products/takano_actuator_p51.pdf

Ellis/Kuhnke Controls – CDR030 Specifications and Cost

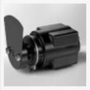

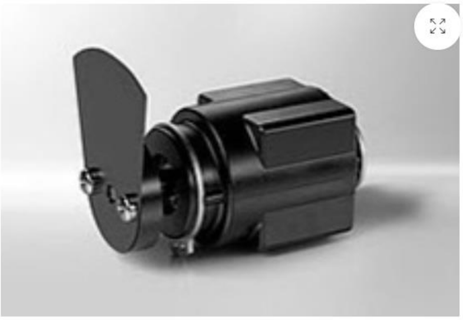

Home / Shop / Optical Shutters / CDR030 Shutter

CDR030 Shutter

$370.90

Based on the bistable rotary solenoid CDR030, Kendrion offers a bistable shutter in the field of optical safety technology that enables currentless holding in both end positions. The built-in permanent magnets hold the end

positions. This makes the magnet design very energysaving.

- 1 + ADD TO CART BUY NOW

Category: Optical Shutters

Technical Data[1]	Shutter CDR030 / bistable
**Supply voltage**	12 and 24 V DC (another voltage on request)
**Diameter magnet**	30 mm
**Rotation angle**	60°, 90° (another rotation angle on request)
**Typical closing time**	30 ms
**Switching frequency**	Up to 30 Hz
**Service life**	20 million switching cycles
**Operating temperature**	-5 °C… +35 °C
**Blade material**	Black anodized aluminium
**Mounting**	Clamp, flanges (detail information available)

[1] We reserve the rights of modification, omission, error with respect to the products. Illustrations similar. All rights reserved by the individual copyright holders.

Link to Datasheet:

https://ekci.shop/wp-content/uploads/2021/08/CDR030.pdf
